# Factors associated with the choice of general medicine as a career among Japanese medical students

**DOI:** 10.3402/meo.v21.29448

**Published:** 2016-05-11

**Authors:** Ryuichi Kawamoto, Daisuke Ninomiya, Yoshihisa Kasai, Tomo Kusunoki, Nobuyuki Ohtsuka, Teru Kumagi, Masanori Abe

**Affiliations:** 1 Department of Community Medicine, Ehime University Graduate School of Medicine, Ehime, Japan; 2 Department of Internal Medicine, Seiyo Municipal Nomura Hospital, Ehime, Japan

**Keywords:** career choice, general medicine, family medicine, Japanese medical students

## Abstract

**Background:**

In Japan, there is a shortage of young physicians in various specialties; the present situation of general medicine or family medicine (GM/FM) in particular is risky. The factors influencing the career choice of Japanese medical students are poorly understood. This study aims to identify factors related to choosing GM/FM as a career.

**Methods:**

The study was designed as a cross-sectional survey. Students at one medical school in Japan filled out a questionnaire. Students were asked to state their intended medical specialty, and they rated the importance of specific individual and occupational aspects using a 4-point likert scale. Factor analysis was performed on the variables. Reliability of the factor scores was estimated using Cronbach‘s alpha coefficients; biserial correlations between the factors and career choices were calculated. Furthermore, multiple linear regression analysis was performed using career choice (GM/FM vs. others) as the criterion variable and the factors plus demographic characteristics as confounding variables.

**Results:**

Factor analysis produced six factors that explained future career plans. Medical students in this study had a positive and realistic idea about GM/FM, but only 18.8% of them chose GM/FM first as a career. The significant variables associated with choosing GM/FM first as a career were: ‘Admission from hometown’ (β=0.189, *P=*0.001), ‘Student preparing for the entrance exam’ (β=0.172; *P=*0.001), ‘Intent for rural practice’ (β=0.123, *P=*0.016), and ‘Work–life balance’ (β=0.126, *P=*0.013). While significant variables that were negatively associated with choosing GM/FM were ‘Presence of medical relatives’ (β=−0.107, *P=*0.037) and ‘Scientific orientation’ (β=−0.125, *P=*0.013).

**Conclusions:**

Strategies have been suggested, such as recruiting medical students with significant variables that were associated with choosing GM/FM first as a career. By engaging students early in their choice of career, we may be able to increase enthusiasm for this specialty.

In Japan, there have been absolute and relative deficiencies in the number of physicians, and the number of those belongs to the lowest group in the Organisation for Economic Co-operation and Development (OECD) (1). There are 80 medical schools – 43 national, 8 prefectural (i.e., founded by a local government), and 29 private – representing approximately one school for every 1.6 million people, and there were 46,610 medical students in 2009; 32.2% were women. After 6 years of medical school, 2 years of clinical training, during which the final decision of career choice is made, is mandatory, and a doctor-to-facility matching system was introduced in 2004. Since then, more young doctors have migrated from academic (university) hospitals to non-academic hospitals (2). Due to the absence of regulatory mechanisms whereby a balanced distribution of workforce is generated, this misdistribution in various facilities and specialties is also noticeable as few doctors opt for specialties that cover broad domains such as internal medicine, surgery, obstetrics and gynecology, pediatrics, and emergency medicine (2). In addition, the misdistribution of physicians between urban and rural areas is increasing remarkably (3). Moreover, aging is progressing at an incomparable speed inexperienced by the world, and the extensive use of community hospitals by patients in Japan has contributed much to the accelerating shortage of physicians.

In the misdistribution and shortage of physicians, communities need doctors who can cater flexibly to the demands from the health care and welfare systems, as is the case for general medicine (GM) in Europe and family medicine (FM) in North America. In 2007, Japan's Ministry of Education, Culture, Sports, Science, and Technology revised the model core curriculum by adopting a clinical training program in communities (4). Thus, community-based education was established as a mandatory education program. Even though the need for such a program has been noticeable for more than 30 years in Japan, it has not yet been established; however, a decision has been made to introduce it in 2017.

Thus, it is important to know the expectations of future physicians as they play a role in their choice of career. There are many studies on career choice of medical students in Western countries. Wright et al. (5, 6) demonstrated that the reasons influencing the career choice of medical students are complex. Previous studies have shown that factors associated with choosing GM/FM as a career are older age (7, 8), gender (being a female) (9), being married (7), rural background (e.g., rural origin and rural high school graduation) (7, 10), parents’ socioeconomic status (7), lifestyle considerations (11, 12), working hours (13, 14), low-income expectations (7, 15–17), lower prestige (7, 15–17), lower job-related ambition (15), intent for rural practice (7), longitudinal and close relationship with patients (9, 11, 12, 16, 17), no plan for a career in research (7, 17), presence of a FM role model (7, 16, 18), and social need (16). More students might consider careers in primary care if they were exposed to more experiences in primary care (5, 9, 16, 19).

On the contrary, there are few studies that demonstrate the background factors associated with GM/FM as a career choice among Japanese medical students (20). Ohtaki et al. (20) demonstrated that 89.8% of 3,377 students desired to become clinicians, 79.3% desired to have general clinical ability, and 54.9% had some or much interest in primary care. However, this study did not demonstrate the determinants of medical students to pursue a GM career. The purpose of this study was twofold: to understand what career preferences medical students have at the beginning of medical school and to determine the factors associated with choosing GM/FM as a career.

## Methods

### Participants

The study was designed as a cross-sectional survey. We conducted a survey of 1st to 5th year medical students (*N*=450) from one Japanese regional university school of medicine. A 5-page entry questionnaire was handed out in class within the first 4 weeks of the start of medical school. The study was approved by the ethics committee of the Ehime University Graduate School of Medicine, and informed consent was obtained from all subjects.

### Questionnaire

We used a modified questionnaire enquiring about their specialty preference and to what extent their decision was influenced by a set of given criteria that were developed by Takeda et al. (21). Sociodemographic questions on gender, age, academic year, admission from hometown, type of high school, admission by a special policy directly aimed at increasing rural physicians as one main purpose (*chiiki-waku* in Japanese), student preparing for the entrance exam, presence of medical relatives, and growing up in a rural area were included. Moreover, the participants were asked whether they were willing to work in a rural area.

Participants were asked to specify which of the following 14 medical specialties they intended to pursue: GM/FM, internal medicine subspecialty, surgery, pediatrics, obstetrics/gynecology, psychiatry, anesthesiology, emergency medicine, dermatology, orthopedics, ophthalmology, otolaryngology, urology and radiology, or ‘other’. They were instructed to choose the specialty most preferable to them and also other specialties ‘under consideration’ with no restriction in the number. When ‘other’ was chosen for a non-listed specialty, respondents were asked to specify which discipline they were choosing. They then indicated the degree to which 30 items influenced their choice (Appendix). The subscales ‘characteristics of the specialty’ (10 items), ‘personal experience’ (three items), ‘experience at a medical school or during postgraduate training’ (five items), ‘advice from others’ (four items), and ‘considering future work condition’ (eight items) covered the reasons for choosing a specialty. The subscale response to the influences was rated on a 4-point likert scale (1=not at all, 2=not particularly, 3=fairly well, and 4=extremely well).

### Data analysis

Statistical analysis was performed using IBM SPSS Statistics Version 21 (Statistical Package for Social Science, Chicago, IL). Data are presented as the mean±standard deviation (SD) unless otherwise specified, and in the cases of parameters with non-normal distributions, the data were log-transformed for analysis. The factor analysis was conducted in IBM SPSS using a maximum likelihood method and promax rotation. For each item, we calculated the mean±SD, and items showing ceiling effect (≥3.2) or floor effect (<1.2) were excluded from the analysis. In order to decide the number of factors, a scree plot, which shows the eigenvalues on the y-axis and the number of factors on the x-axis, was generated, and a cutoff of eigenvalue was set to be greater than 1. Item retention was based on coefficient values (factor loadings ≥ 0.35) or showing a similar factor loading in more than two factors were excluded, and then the factor analysis was repeated. We calculated the Cronbach's alpha coefficient for each factor to determine its scale reliability and calculated a mean score and SD. Differences in the subscales by level of interest in choosing GM/FM as a career were examined using ANOVA for continuing variables and *χ* ^2^-test for categorical variables. Finally, stepwise multiple linear regression analysis was performed in order to derive confounding factors associated with level of interest in choosing GM/FM as a career. A *p*-value<0.05 was considered significant.

## Results

### Characteristics of participants

Of 417 students, 368 completed the survey giving an 88.2% response rate. In Table 1, characteristics of participants by level of interest for GM/FM as a career choice are summarized. Most students tended to score higher regarding their intent to choose GM/FM as a career in admission from hometown, public high school graduation, combined junior high and high school graduation, admission by a special policy, and intent for rural practice. Gender, age, academic year, and student preparing for the entrance exam next year, presence of medical relatives, presence of a role model, and growing up in a rural area did not have a significant influence on the choice of GM/FM as a career.

**Table 1:** Characteristics of participants by level of interest in choosing GM/FM as a career

		General physician/family physician, *N* (%)			
			
	Total	Most interested	Interested	Others	
		
Characteristics	*N*=368	*N*=69	*N*=156	*N=*143	*P* for trend
Gender					
Women	141 (38.3)	27 (39.1)	63 (40.4)	51 (35.7)	0.695
Men	227 (61.7)	42 (60.9)	93 (59.6)	92 (64.3)	
Age, years	21.4±3.6	21.3±2.3	21.7±4.0	21.2±3.6	0.538
Academic year					
1st–2nd	179 (48.6)	31 (44.9)	69 (44.2)	79 (55.2)	0.129
3rd–5th	189 (51.4)	38 (55.1)	87 (55.8)	64 (44.8)	
Admission from hometown					
Yes	168 (45.7)	43 (62.3)**	64 (41.0)	61 (42.7)	**0.008**
No	200 (54.3)	**26 (37.7)**	92 (59.0)	82 (57.3)	
Public high school graduation					
Yes	195 (53.0)	46 (66.7) ***	85 (54.5)	64 (44.8)	**0.010**
No	173 (47.0)	**23 (33.3)**	71 (45.5)	79 (55.2)	
Combined junior high and high school graduation					
Yes	164 (44.6)	22 (31.9)**	66 (42.3)	76 (53.1)	**0.011**
No	204 (55.4)	**47 (68.1)**	90 (57.7)	67 (46.9)	
Admission by a special policy					
Yes	23 (6.3)	9 (13.0)*	8 (5.1)	6 (4.2)	**0.033**
No	345 (93.8)	**60 (87.0)**	148 (94.9)	137 (95.8)	
Student preparing for the entrance exam next year					
Yes	175 (47.6)	40 (58.0)	67 (42.9)	68 (47.6)	0.115
No	193 (52.4)	29 (42.0)	89 (57.1)	75 (52.4)	
Presence of medical relatives					
Yes	162 (44.0)	23 (33.3)	72 (46.2)	67 (46.9)	0.139
No	206 (56.0)	46 (66.7)	84 (53.8)	76 (53.1)	
Growing up in a rural area					
Yes	47 (12.8)	10 (14.5)	18 (11.5)	19 (13.3)	0.809
No	321 (87.2)	59 (85.5)	138 (88.5)	124 (86.7)	
Intent for rural practice					
Yes	66 (17.9)	20 (29.0)**	27 (17.3)	19 (13.3)	**0.020**
No	302 (82.1)	**49 (71.0)**	129 (82.7)	124 (86.7)	

*P* for trend from ANOVA for continuing variables and *χ*^2^-test for categorical variables.

* *P<*0.05

** *P<*0.01

*** *P<*0.005 versus ‘other’ groups. Numbers in bold indicate significance (*p<*0.05).

### Factor analysis

The results of the factor analysis including the factor loadings of the items and the internal consistency of the factors (Cronbach's alpha) are shown in Table 2. Some items were excluded from the factor analysis because of the ceiling effect; four because of low factor loading (<0.35); and one because it correlated with two factors to the same extent. We defined the following six factors based on the types of items that grouped together; Factor 1: Educational experience; Factor 2: Job security; Factor 3: Advice from others; Factor 4: Work–life balance; Factor 5: Scientific orientation; and Factor 6: Personal reasons. These six factors collectively accounted for 47.6% of the variance in response. We calculated Cronbach's alpha co-efficiencies which demonstrated internal consistency that ranged between 0.55 and 0.84.

**Table 2:** Factor analysis of specialty preferences associated with future career plans

	Factor structure (Factor loading)					
	
Item	1	2	3	4	5	6
Factor 1: Educational experience (Cronbach's α=0.84)						
15) Received excellent teachings	0.92	0.13	−0.12	−0.06	0.06	−0.06
14) Memorable experience at a class or clinical rotation	0.79	0.10	−0.08	−0.08	0.02	0.13
16) Comfortable atmosphere at the specialty department	0.77	−0.11	0.02	0.16	−0.10	−0.01
17) Encounter with role model teachers	0.56	−0.19	0.26	0.08	−0.03	−0.01
Factor 2: Job security (Cronbach's α=0.73)						
19) Advice/expectation of parents	0.05	0.59	0.11	−0.19	−0.09	0.07
26) Expected income	0.01	0.58	−0.06	0.32	0.07	0.09
24) Ease of opening a practice	−0.10	0.54	−0.01	0.19	0.20	0.04
25) Expectation to inherit the practice of my parents/relatives	−0.09	0.50	−0.00	0.03	−0.13	0.11
23) Job availability	0.18	0.46	0.10	0.09	0.04	−0.06
Factor 3: Advice from others (Cronbach's α=0.81)						
20) Advice from senior students/residents	−0.04	0.04	0.84	0.01	−0.12	0.04
21) Advice from teachers/consultants	0.07	−0.02	0.81	0.01	0.05	−0.05
22) Influence of friends	−0.08	0.16	0.57	0.04	−0.01	0.04
Factor 4: Work–life balance (Cronbach's α=0.78)						
27) Working hours	−0.02	0.02	0.03	0.84	−0.01	−0.07
28) Attainable lifestyle	0.06	0.04	−0.02	0.67	0.11	0.01
30) Risk of any malpractice law suits	0.04	0.16	0.10	0.52	−0.06	0.02
Factor 5: Scientific orientation (Cronbach's α=0.70)						
5) Interest in surgical procedures or technologies	−0.08	0.13	0.08	−0.21	0.77	−0.14
6) Mastering a specialty	0.00	−0.08	−0.00	0.04	0.62	−0.02
4) Interest in research or scientific aspects	−0.00	−0.10	−0.03	0.16	0.60	0.24
2) Interest in organ specialty	0.05	−0.07	−0.03	0.11	0.48	0.04
Factor 6: Personal reasons (Cronbach's α=0.55**)**						
11) I suffer(ed) from an illness in that specialty	−0.11	0.02	−0.09	0.15	−0.03	0.56
12) Friend/family suffer(ed) from an illness in that specialty	−0.02	0.10	0.09	−0.09	0.01	0.56
13) Became interested in the specialty before medical school	0.12	0.12	−0.02	0.15	−0.01	0.42
3) Interest in targeted populations such as children or the elderly;	0.12	−0.13	0.07	−0.09	0.11	0.38
Factor: Inter-factor correlations	1	2	3	4	5	6
1	1.00					
2	0.16	1.00				
3	0.29	0.46	1.00			
4	0.25	0.53	0.47	1.00		
5	0.08	−0.01	0.03	0.88	1.00	
6	0.22	0.18	0.20	0.09	0.12	1.00

The factor analysis was conducted with IBM SPSS using a maximum likelihood method and promax rotation. Items excluded from the factor analysis because of a ceiling effect were: 1) Interest in clinical work of the specialty and 8) I feel it rewarding to work in the specialty, low factor loading (<0.35); 7) I have an aptitude for the specialty; 9) Prospect for further development of the field; 10) Highly respected in society; and 29) Influence of future health care reform, correlation with two factors to the same extent; 18) Encounter with a role model junior doctor

### Relationship between confounding factors and choosing GM/FM as a career

In Table 3, characteristics including factors of specialty preferences associated with future career plans are summarized in relation to level of interest in choosing GM/FM as a career. Pearson's correlation coefficient between confounding factors and choosing GM/FM as a career showed that students choosing GM/FM first as a career were more likely to be in the ‘admission from hometown’, ‘Public high school graduation’, ‘Admission by a special policy’, and ‘Intent for rural practice’ groups, but less likely to be in the ‘Combined junior high and high school graduation’, ‘Presence of medical relatives’, and ‘Scientific orientation’ groups. In addition, stepwise multiple linear regression analysis using level of interest in choosing GM/FM as a career as an objective variable, adjusted for confounding factors as the explanatory variable, showed that ‘Academic year’, ‘Admission from hometown prefecture’, ‘Public high school graduation’, ‘Student preparing for the entrance exam’, ‘Admission by a special policy’, ‘Intent for rural practice’, and ‘Work–life balance’ were independently and positively associated with choice as a career. ‘Presence of medical relatives’ and ‘Scientific orientation’, on the contrary, were less interesting for students aiming for GM/FM.

**Table 3:** Relationship between confounding factors and level of interest in choosing GM/FM as a career

	1st choice=1 and 2nd choice+others=0 *N*=368		1st choice=2, 2nd choice=1 and others=0 *N*=368	
		
Characteristics	*r* (*p*-value)	β (*p*-value)	*r* (*p*-value)	β (*p*-value)
Gender (Men: 1, Women: 0)	−0.008 (0.878)	–	−0.033 (0.525)	–
Age	−0.020 (0.695)	–	0.017 (0.740)	–
Academic year	0.036 (0.495)	–	0.089 (0.088)	**0.120 (0.023)**
Admission from hometown	**0.161 (0.002)**	**0.189 (0.001)**	**0.188 (0.024)**	–
Public high school graduation	**0.132 (0.011)**	–	**0.158 (0.002)**	**0.157 (0.002)**
Combined junior high and high school graduation	−**0.123 (0.019)**	–	−**0.157 (0.003)**	–
Admission by a special policy	**0.135 (0.010)**	–	**0.117 (0.025)**	**0.105 (0.043)**
Student preparing for the entrance exam	0.100 (0.055)	**0.172 (0.001)**	0.053 (0.306)	–
Presence of medical relatives	−**0.103 (0.047)**	−**0.107 (0.037)**	−0.045 (0.384)	–
Growing up in a rural area	0.025 (0.636)	–	−0.012 (0.814)	–
Intent for rural practice	**0.138 (0.008)**	**0.123 (0.016)**	**0.138 (0.008)**	**0.128 (0.014)**
Future career plans				
Educational experience	0.054 (0.300)	–	0.050 (0.340)	–
Job security	0.037 (0.483)	–	0.041 (0.438)	–
Advice from others	0.056 (0.288)	–	0.028 (0.593)	–
Work–life balance	0.092 (0.079)	**0.126 (0.013)**	0.067 (0.199)	–
Scientific orientation	−0.098 (0.061)	−**0.125 (0.013)**	−**0.136 (0.009)**	−**0.116 (0.024)**
Personal reasons	0.025 (0.629)	–	0.034 (0.521)	–
*R* ^2^	–	**0.085 (<0.001)**	–	**0.082 (** ***p<*** **0.001)**

*r*, Pearson's correlation coefficient; β, standardized coefficient; *R* ^2^, coefficient of determination. ‘–’ was not retained in the final model by multiple linear regression analysis (stepwise method). Numbers in bold indicate significance (*p<*0.05).

## Discussion

The purpose of this study was to determine factors that are associated with level of interest in choosing GM/FM as a career among Japanese medical students. Some factors found to be important for making this choice in previous studies were also found important in this study. Students highly influenced by the factors of ‘Academic year’, ‘Admission from hometown’, ‘Public high school graduation’, ‘Student preparing for the entrance exam’, ‘Admission by a special policy’, ‘Intent for rural practice’, and ‘Work–life balance’ were more likely to choose GM/FM as a career. Both ‘Presence of medical relatives’ and ‘Scientific orientation’ did not influence the choice of GM/FM as a career. These findings suggest that students’ characteristics may be strongly associated with choosing GM/FM as a career, and more research is needed to examine the effectiveness of specific educational interventions in promoting GM/FM as a career choice.

In our study, some positive and negative factors that were significantly and independently associated with the choice of GM/FM as a career were similar to those in the Western reports. Medical students in a higher academic year of training are more likely to choose the GM/FM career. In Japan, all medical schools including ours provide education on community-based medicine during the 3rd to 4th academic year. Senf et al. (7) demonstrated that mandatory FM time in the 3rd or 4th year is positively related to higher numbers of students selecting FM. Several cases where experiences in medical school tend to relate to the choice of specialty and the presence of faculty role models in medical schools are very important as they serve both as positive and negative influences (22). Therefore, it is important for medical students to meet family physicians as role models. Special programs for primary care are also important for higher proportions of graduates in FM from the special pathway than from the conventional curriculum (7).

In the present study, ‘Public school graduation’ and ‘Admission from hometown’ were significantly associated with the choice of GM/FM as a career. In Japan, ‘Private high school graduation’, ‘Combined junior high and high school graduation’ and ‘Presence of medical relatives’ are related to a higher socioeconomic status. There are some studies showing that the higher socioeconomic status of parents relates negatively to the choice of FM (7, 23). Medical students who graduated from a public high school in their hometown have low-income expectations and believe that primary care is important (7). The present study suggests that medical schools might recruit students with such a mindset in order to increase the number of aspirants of GM/FM.

‘Intent for rural practice’ is important for choice of FM/GM as a career. Medical students understand that career choice of FM/GM is necessary for rural practice which is expected to cover a broad range of conditions. Rabinowitz et al. (24) demonstrated that students with an interest in FM who enter medical school are more likely than their peers to become family physicians or practice in a rural area. ‘Admission by a special policy’ is a system whereby students can enter a medical school on the condition that they remain and work in a local prefecture after graduation. Moreover, programs such as debt cancellation of student loans may be useful in recruiting students to rural practice (25), and the career choice of GM/FM.

In our study, the factor of ‘Work–life balance’, which consists not only of ‘Working hours’ and ‘Attainable lifestyle’ but also ‘Risk of my malpractice law suits’ was also significantly and independently associated with choosing GM/FM first as a career. Newton et al. demonstrated that lifestyle as well as income has become more important to medical students in their career choice (26). Students perceived the GM/FM specialties as lifestyle intermediate compared to other specialties (24, 26, 27). In our study, the career choice of GM/FM was significantly related to ‘Work–life balance’. A report from Germany confirms that future general physicians differ from students intending to choose other specialties, particularly in terms of individual aspects such as ‘Work–life balance’ (11).

Scientific orientation is a background characteristic that was negatively associated with choice of GM/FM as a career. ‘Interest in the surgical procedures or technologies’, ‘Mastering the specialty’, and ‘Interest in an organ specialty’ which identify with ‘Scientific orientation’ are strongly related to organ-specific specialty and not important for students selecting GM/FM. Medical students with less interest in research or scientific aspects do not plan a research career. This tendency is consistent with that of previous studies in Western countries, and for that reason, it is possible that, compared with other specialties of physicians, many family physicians do not serve as good research role models (28). Then, strategies and initiatives intended to increase research within the specialty of FM should be evaluated for their effects on student career choices (29).

Some limitations of this study must be considered. First, our cross-sectional study design does not eliminate potential causal relationships between characteristics of medical students and career choice. Second, this study was on a limited number of students who belong to one local university. Therefore, the students were more likely to choose GM/FM than students from other universities. Third, our study measured students’ interest in the GM/FM career, but not their actual choice of practice because students’ intent was measured prior to their residency. Fourth, as we used a self-administered questionnaire developed for 1st to 5th year medical students, some of the characteristics examined appeared to be suitable for upper grade students (such as choice of specialty) but not for lower grade students. Future research using longitudinal data collection will enable us to monitor the relationship between early stated interest and actual behavior.

## Conclusion

The present study showed that medical students have a positive and realistic picture of what general practice is all about; however, only 18.8% of students in the study ranked GM/FM as their first choice career. They highlighted that the important factors considered when choosing a future career were: ‘Intent for rural practice’ and ‘Fulfilling lifestyle’, while ‘Presence of medical relatives’ and ‘Scientific orientation’ did not seem to be of much importance. Strategies have been suggested, such as redesigning the curriculum to give students more exposure to the diversity of general practice. By engaging students early in their career, we may be able to increase their enthusiasm for this specialty.

## Appendix

**Table:** To what extent do the following reasons for choosing a specialty match your own? Please circle one response for each factor.

(4. extremely, 3. fairly well, 2. not particular, 1. not at all)				
1. Interest in clinical work of the specialty:	4	3	2	1
2. Interest in an organ of the specialty	4	3	2	1
3. Interest in the targeted population (e.g., children, the elderly)	4	3	2	1
4. Interest in research or scientific aspects	4	3	2	1
5. Interested in surgical procedures or technologies	4	3	2	1
6. Mastering the specialty	4	3	2	1
7. I have an aptitude for the specialty	4	3	2	1
8. I feel it rewarding to work in the specialty	4	3	2	1
9. Prospect for further development of the field	4	3	2	1
10. Highly respected in society	4	3	2	1
11. I suffer(ed) from an illness of the specialty	4	3	2	1
12. Friend/family suffer(ed) from an illness of the specialty	4	3	2	1
13. Because I was interested in the specialty before medical school	4	3	2	1
14. Memorable experience at a class or clinical rotation	4	3	2	1
15. Received excellent teaching	4	3	2	1
16. Comfortable atmosphere at the specialty department	4	3	2	1
17. Encounter with role model teachers	4	3	2	1
18. Encounter with role model junior doctors	4	3	2	1
19. Advice/expectation of parents	4	3	2	1
20. Advice from senior students/residents	4	3	2	1
21. Advice from teachers/consultants	4	3	2	1
22. Influence of friends	4	3	2	1
23. Job availability	4	3	2	1
24. Ease of opening a practice	4	3	2	1
25. Expectation to inherit a practice of my parents/relatives	4	3	2	1
26. Expected income	4	3	2	1
27. Working hours	4	3	2	1
28. Attainable lifestyle	4	3	2	1
29. Influence of future health care reforms	4	3	2	1
30. Risk of malpractice law suits	4	3	2	1
